# Tunable Transmissive Terahertz Linear Polarizer for Arbitrary Linear Incidence Based on Low-Dimensional Metamaterials

**DOI:** 10.3390/nano11071851

**Published:** 2021-07-18

**Authors:** Zhenyu Yang, Dahai Yu, Huiping Zhang, Anqi Yu, Xuguang Guo, Yuxiang Ren, Xiaofei Zang, Alexei V. Balakin, Alexander P. Shkurinov

**Affiliations:** 1 Shanghai Key Lab of Modern Optical System, Terahertz Spectrum and Imaging Technology Cooperative Innovation Center, Terahertz Technology Innovation Research Institute, University of Shanghai for Science and Technology, 516 Jungong Road, Shanghai 200093, China; 192380304@st.usst.edu.cn (Z.Y.); hpzhang@usst.edu.cn (H.Z.); xgguo@usst.edu.cn (X.G.); 182390295@st.usst.edu.cn (Y.R.); xfzang@usst.edu.cn (X.Z.); a.v.balakin@physics.msu.ru (A.V.B.); ashkurinov@physics.msu.ru (A.P.S.); 2 Focused Photonics (Hangzhou) Inc., No: 760, Bin’an Road, Binjiang District, Hangzhou 310052, China; dahai_yu@fpi-inc.com; 3 Shanghai Institute of Intelligent Science and Technology, Tongji University, Shanghai 200092, China; 4 Faculty of Physics and International Laser Center, Lomonosov Moscow State University, Leninskie Gory 1-2, 19991 Moscow, Russia; 5 ILIT RAS–Branch of the FSRC “Crystallography and Photonics” RAS, Svyatoozerskaya 1, 140700 Shatura, Russia

**Keywords:** terahertz, graphene, plasmons, Drude absorption, polarization conversion

## Abstract

In this work, we propose a structure consisting of three metamaterial layers and a metallic grating layer to rotate the polarization of arbitrary linearly polarized incidence to the y-direction with high transmissivity by electrically tuning these metamaterials. The transfer matrix method together with a harmonic oscillator model is adopted to theoretically study the proposed structure. Numerical simulation based on the finite difference time-domain method is performed assuming that the metamaterial layers are constituted by graphene ribbon arrays. The calculation and simulation results show that the Drude absorption is responsible for the polarization rotation. Fermi level and scattering rate of graphene are important for the transmissivity. For a polarization rotation of around 90°, the thickness of either the upper or lower dielectric separations influences the transmission window. For a polarization rotation of around 45° and 135°, the lower dielectric separations decide the frequency of the transmission window, while the upper dielectric separations just slightly influence the transmissivity.

## 1. Introduction

Terahertz waves, electromagnetic waves with frequencies ranging from 0.1 THz to 10 THz, have extraordinary characteristics such as being non-ionizing, having a large bandwidth, and molecular fingerprints, which offer terahertz waves potential applications in medical examination, high-speed communication, environmental monitoring, etc. These applications require terahertz sources, detectors, and other functional devices such as polarization rotators or wave plates with high performances. Conventionally, polarization rotators use birefringence [1,2], total internal reflection effects [3,4], and the Faraday effect [5,6] to change the polarization. However, birefringent rotators are bulky, total internal reflection rotators need complex designs and fabrication, and Faraday rotators require the application of an external magnetic field.

In recent years, metamaterial-based polarization rotators have attracted a lot of attention as metamaterials can realize birefringence with a total device thickness less than the incident light. For transmissive polarization rotators, polarization rotation is usually achieved by deliberate design of the metamaterial to tune the phase and the amplitude of the two orthogonal components [7,8,9,10,11,12,13,14,15]. However, since the phase difference and the amplitude of two orthogonal components change as the frequency of the incidence changes, the bandwidths of such transmissive polarization rotators are usually limited [7,8,9,10,11,12,13,14]. Metallic gratings can help to enhance the bandwidth and the transmissivity [16,17,18,19,20,21,22]. Electromagnetic waves transmitted through or reflected by the metamaterials with unwanted polarization are reflected by metallic gratings and interact with the metamaterials again, so the transmission window grows taller and wider.

As the material properties of dielectrics and metals can hardly be tuned by an applied electric field, polarization rotators based on these metamaterials lack post-fabrication tunability. This weakness can be compensated by low-dimensional materials, e.g., graphene. The tuning on the phase and amplitude caused by graphene plasmons or Drude absorption (which is considered as 0th order plasmon resonance) can be actively tuned by the graphene Fermi level, offering the opportunity to realize actively tunable polarization rotators. Recently, Zhang et al. combined two metallic gratings and two orthogonal graphene gratings to actively tune the polarization of the transmitted waves from 20° to 70° by changing the Fermi level on each graphene grating [23]. Qi et al. demonstrated a reflective quarter-wave plate and half-wave plate by changing the graphene Fermi level [24]. Peng et al. proposed a reflective half wave-plate whose functioning frequency can be tuned by changing the graphene Fermi level [25]. Although there has been a plethora of simulation works realizing polarization conversion, little has been done on theoretical models which can explain the influence of physical and structural parameters on the efficiency of the polarization conversion, yield good correspondence with simulated results, and offer quick optimization of the proposed structures, especially for transmissive polarization converters.

In this work, we propose an actively tunable polarization converter consisting of a metamaterial layer (MML) on the top, a metallic grating layer in the bottom, and two MMLs in between, to realize electrically tunable polarization conversion in linearly polarized transmission for arbitrary linearly polarized incidence with high transmission efficiency. A theoretical model with the abovementioned functionalities is constructed by the transfer matrix method (TMM). Then, we numerically simulate the structure by the finite difference time-domain (FDTD) method by assuming that the MMLs are constituted by graphene ribbon arrays. Fermi level and the scattering rate of graphene are important for the efficiency of the polarization rotation. In particular, the 1^st^ MML functions well as a linear polarizer only when the high carrier density is high, which is vital for efficient rotation angles around 90°. This result may find applications in THz communication, imaging, manipulation, etc.

## 2. Model Descriptions

**Figure 1:**
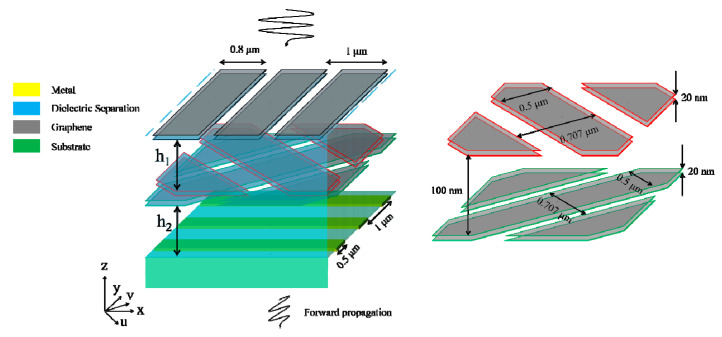
Schematics of the proposed structure.

The proposed structure is schematically shown in Figure 1. The 1st MML is assumed to have resonances along the *y*-coordinate. The 2nd and 3rd MMLs in the middle are assumed to have resonances along the *u*-coordinate and *v*-coordinate, respectively, with $\vec{u}=\frac{1}{\sqrt{2}}(\vec{x}-\vec{y})$ and $\vec{v}=\frac{1}{\sqrt{2}}(\vec{x}+\vec{y})$. The metallic gratings in the bottom extend along the *x*-coordinate and function as a linear polarizer, allowing only the transmission of *y*-polarized electromagnetic waves and reflecting the *x*-polarized waves. The 2nd and 3rd MMLs are separated from the 1st MML and the metallic gratings by an upper dielectric layer with thickness *h*_1_ and a lower dielectric layer with thickness *h*_2_. The refractive indices of the two dielectric layers and the substrate are all *n*_1_ for simplicity. The boundary matrix *T_top_* at the surface of the proposed structure can be calculated as:

(1) $$T_{top}=\left(\begin{array}{l} E_{x}^{+}\\ E_{x}^{-}\\ E_{y}^{+}\\ E_{y}^{-} \end{array}\right)=M_{top}M_{upper}M_{uv\to xy}M_{res}M_{xy\to uv}M_{lower}T_{bottom},$$

where

(2) $$M_{res}=\frac{1}{2}\left(\begin{array}{cccc} 1+\frac{n_{2}}{n_{1}}+\frac{\sigma_{u}\left(\omega \right)}{\varepsilon_{0}c} & 1-\frac{n_{2}}{n_{1}}+\frac{\sigma_{u}\left(\omega \right)}{\varepsilon_{0}c} & 0 & 0\\ 1-\frac{n_{2}}{n_{1}}-\frac{\sigma_{u}\left(\omega \right)}{\varepsilon_{0}c} & 1+\frac{n_{2}}{n_{1}}-\frac{\sigma_{u}\left(\omega \right)}{\varepsilon_{0}c} & 0 & 0\\ 0 & 0 & 1+\frac{n_{2}}{n_{1}}+\frac{\sigma_{v}\left(\omega \right)}{\varepsilon_{0}c} & 1-\frac{n_{2}}{n_{1}}+\frac{\sigma_{v}\left(\omega \right)}{\varepsilon_{0}c}\\ 0 & 0 & 1+\frac{n_{2}}{n_{1}}-\frac{\sigma_{v}\left(\omega \right)}{\varepsilon_{0}c} & 1+\frac{n_{2}}{n_{1}}-\frac{\sigma_{v}\left(\omega \right)}{\varepsilon_{0}c} \end{array}\right),$$

is the transfer matrix describing the 2nd and 3rd MMLs by considering them as a whole [22]. *σ_u_(ω)* and *σ_v_(ω)* are the effective conductivities in the *x*- and *y*-coordinates, respectively, *ε*_0_ is the dielectric constant in vacuum, and *c* is the speed of light in vacuum. *M_upper_* and *M_lower_* are the transfer matrices of the upper and lower dielectric separations, *M_uv_*_→*xy*_ and *M_xy_*_→*uv*_ are the transfer matrices describing the coordinate transformation, *M_top_* is the transfer matrix describing plasmon resonances of the 1st MML at the surface of the proposed structure, and *T_bottom_* describes the electric field at the upper surface of the metallic gratings. The details of these transfer matrices and boundary matrix have been given in our previous work [22]. Here, the spacing between the 2nd and 3rd MMLs (~100 nm) is ignored because it is far less than the relevant wavelength (~ 300 μm).

In our previous work [22], it was pointed out that plasmon resonances or Drude absorption should not be excited simultaneously with the same frequency and strength in the *u*- and *v*-coordinates. Therefore, the 2nd and 3rd MMLs cannot be in the “ON” state (the MML is electrically biased and has plenty of charge carriers) simultaneously. When the 1st MML is in the “ON” state, it is supposed to function as a linear polarizer, allowing only the transmission of *x*-polarized electromagnetic waves and reflecting the *y*-polarized waves. Then, the proposed structure functions differently as the state of the 1st MML changes. If the 1st and the 2nd/3rd MML are in the “ON” state, the *x*-component of the incidence will pass through the 1st MML. Then, the change in the amplitude and phase induced by the plasmon resonance or Drude absorption of the 2nd/3rd MML will change the polarization of the electromagnetic waves, generating the *y*-component by transforming the *x*-polarized waves into elliptically polarized waves. When the elliptically polarized waves in the lower dielectric separation arrives at the metallic gratings, the *y*-component will pass through while the *x*-component will be reflected. The reflected *x*-polarized waves will again be transformed into elliptically polarized waves, whose *y*-component will be reflected back into the cavity by the 1st MML. The total transmission will be enhanced if constructive interaction takes place in the cavity formed between the 1st and the 2nd/3rd MML and the cavity formed between the 2nd/3rd MML and the metallic gratings. In this case, the proposed structure is similar to the one studied in our previous work [22], and polarization rotation around 90° is anticipated. However, there remains the problem of how well the 1st MML works as a linear polarizer, which is important for the efficiency of the cross-polarization conversion. Due to the fact that the model is too complicated and a clear conclusion can hardly be drawn, in this section, we mainly focus on the theoretical study of the second case, that is, when the 1st and the 3rd MMLs are in the “OFF” state (the MML is not electrically biased and has few charge carriers), while the 2nd MML is in the “ON” state. In the second case, the *v*-component can pass through the 2nd MML with little loss, while the *u*-component can hardly pass through. The *v*-component can be divided into *x*- and *y*-components with equal amplitude, with the former reflected back by, and the latter passing through, the metallic gratings. Then, the reflected *x*-component is divided into *u*- and *v*-components, with the former strongly reflected by the 2nd MML. The total transmission will be enhanced if constructive interaction takes place in the cavity formed between the 2nd MML and the metallic gratings. Then, it is anticipated that the polarization conversion is most efficient for incidence polarized along the v-coordinate because there is no reflection when the incidence arrives at the 2nd MML for the first time.

Normalizing the transmitted energy as 1, the amplitude of the incidence is calculated as:

(3) $$E_{v}^{+}=\frac{e^{-ik_{0}n_{1}\left(h_{1}+h_{2}\right)}}{2\sqrt{2}}\left[\frac{1+n_{1}+\frac{\sigma_{u}(\omega )}{\varepsilon_{0}c}e^{ik_{0}n_{1}h_{1}}B}{1-i\frac{\sigma_{u}(\omega )}{\varepsilon_{0}c}\sin\left(k_{0}n_{1}h_{2}\right)\frac{B}{A}}+1+n\right]$$

(4) $$A=n_{1}\cos[k_{0}n_{1}\left(h_{1}+h_{2}\right)]-i\sin[k_{0}n_{1}\left(h_{1}+h_{2}\right)]$$

(5) $$B=n_{1}\cos\left(k_{0}n_{1}h_{1}\right)-i\sin\left(k_{0}n_{1}h_{1}\right)$$

where *k*_0_ is the wavevector of the incidence in the vacuum. Mathematically, |*E_v_*^+^| should be small enough to achieve high transmissivity, so the numerator in the square bracket in Equation (3) should be small while the denominator should be large. Note that |*σ_u_(ω)/ε_0_c*| >> 1 if the plasmon resonance or the Drude absorption in the *u*-coordinate is strong, then the denominator is possibly dominated by the 2nd term. Since there is “sin(*k_0_n_1_h*_2_)”, a large denominator requires that sin(*k_0_n_1_h*_2_) cannot be 0. Assuming that *k*_0_*n*_1_*h*_2_ is an odd multiple of π/2, |B/A| achieves the maximum value of *n*_1_ when *k*_0_*n*_1_*h*_1_ is an even multiple of π/2. Although the 3rd term in the numerator also achieves the maximum value if *k*_0_*n*_1_*h*_1_ is an even multiple of π/2, the denominator benefits more than the numerator, so the transmissivity is enhanced under this condition. Undoubtedly, *h*_2_ plays a much more important role that *h*_1_ does. Other important factors affecting the transmissivity, according to our previous work [22], may include *E_F_* and the scattering rate.

## 3. Simulation Results and Discussion

To verify the theoretical analyses, we perform FDTD simulations with Lumerical FDTD Solutions. The metal is modeled as perfect electric conductor (whose dielectric function is treated as 1 + 10^6^i in the software with i the imaginary unit) with 100 nm thickness. MMLs are assumed to be paired graphene ribbon arrays (GRAs) such that they can gate themselves. Graphene is characterized by the Kubo formula [23] and the scattering rate is initially set as 10^12^ Hz. The thickness of graphene is set as 1 nm, and the minimum meshes at the graphene boundaries are set as 0.1 nm to ensure the accuracy. The widths of the 1st GRA pair, the 2nd/3rd GRA pairs, and the bottom metallic gratings are 0.8 μm, 0.5 μm, and 0.5 μm, respectively, and the periods are 1 μm, 0.707 μm, and 1 μm, respectively. *n*_1_ is set as 1.4, *h*_1_ and *h*_2_ are initially set as 56 μm. For the given parameters, *k_0_n_1_h*_2_ = mπ/2 with m being an integer yields f_0_ ≈ m*0.957 THz with f_0_ being the frequency of the incidence. In the theoretical calculation, for the 1st, the 2nd, and the 3rd GRA pairs, Drude absorption is included only in *σ_y_*(*ω*), *σ_u_*(*ω*), and *σ_v_*(*ω*), respectively, while high-order plasmon resonances are excluded.

**Figure 2:**
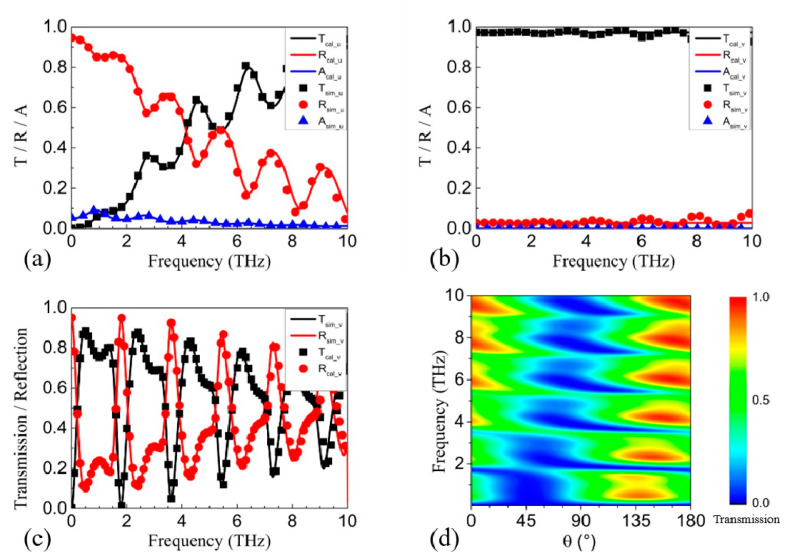
The calculated and simulated transmission, reflection, and absorption spectra for incidence polarized along (**a**) the *u*-coordinate and (**b**) the *v*-coordinate in the absence of the 1st GRA pair, the 3rd GRA pair, and the metallic gratings. (**c**) The calculated and simulated transmission spectra for incidence polarized along the *v*-coordinate with the 1st and 3rd GRA pairs in the “OFF” state. (**d**) The contour by rotating θ from 0° to 180° with the 1st and 3rd GRA pairs in the “OFF” state.

Firstly, we keep the 1st and 3rd GRA pairs in the “OFF” state (*E_F_* = 0 eV) and the 2nd pair in the “ON” state (*E_F_* = 0.9 eV). Figure 2a,b show the calculated and simulated transmission, reflection, and absorption spectra for incidence polarized along the *u*-coordinate and the *v*-coordinate, respectively, in the absence of the metallic gratings. As shown in Figure 2a, the reflection/transmission is very high/low at the low frequencies, and gradually falls/rises as the frequency increases. The peaks and dips result from the resonances in the Fabry–Pérot-like cavity formed by the surface of the proposed structure and the 2nd GRA pair. Obviously, *u*-polarized incidence with a frequency around 1 THz can hardly pass through the 2nd GRA pair. For *v*-polarized incidence, the reflection/transmission is nearly 0%/100%, as shown in Figure 2b, which means that *v*-polarized incidence can pass through the 2nd GRA pair with little loss. The slight fluctuation in the simulated spectra, which is not observed in the calculated spectra, is probably caused by the plasmon resonances in the *v*-coordinate above 10 THz. The excellent correspondence between the calculated and simulated spectra below 4 THz shows that the treatment on *σ_u_(ω)* and *σ_v_(ω)* works well in the concerned frequency range. Since *u*-polarized incidence is blocked while *v*-polarized incidence passes through, the polarization conversion is first studied for *v*-polarized incidence (that is, in the presence of the metallic gratings). As shown in Figure 2c, both the calculated and the simulated transmission spectra show high transmission at odd multiples of 0.93 THz and low transmission in close vicinity to even multiples of 0.93 THz, corresponding to the calculated 0.957 THz. The correspondence between the theoretical analysis and the transmission spectra demonstrates the effectiveness of our model. In our previous work [22], it was shown that either Drude absorption or plasmon resonances can tune the phase and amplitude of the incidence with polarization in the same coordinate. As there is only Drude absorption in the *u*-coordinate and no plasmon resonance in either the *u*- or *v*-coordinate, the *u*-directional Drude absorption is the cause of the polarization conversion. Since both the real part and the imaginary part of the Drude absorption become smaller as the frequency increases, the ability of the polarization conversion gets weaker, and then the transmission windows become weak as m increases. The polarization of the incidence is then rotated by θ from 0° to 180°, with θ representing the counterclockwise angle from the y-axis to the polarization direction. As shown in Figure 2d, the transmission around 0.93 THz exceeds 70% for θ = 115° ~ 160°.

**Figure 3:**
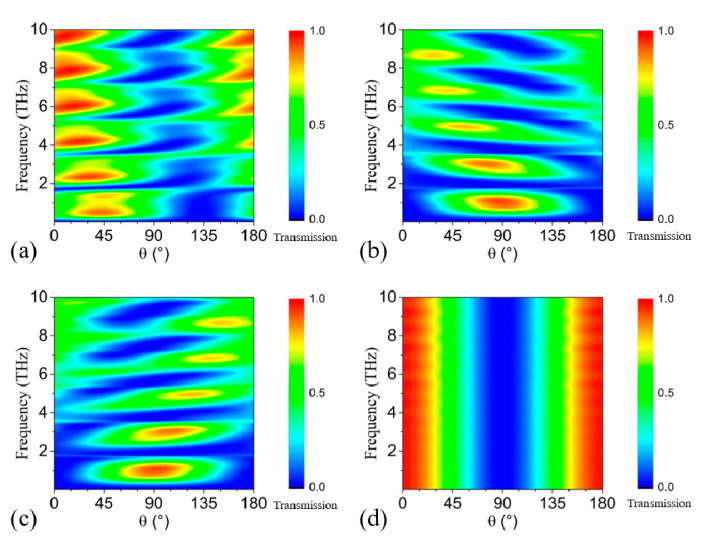
(**a**) The contour by rotating θ from 0° to 180° with (**a**) the 1st and 2nd GRA pairs in the “OFF” state, (**b**) the 2nd GRA pair in the “OFF” state, (**c**) 3rd GRA pair in the “OFF” state, (**d**) all the GRA pairs in the “OFF” state.

**Figure 4:**
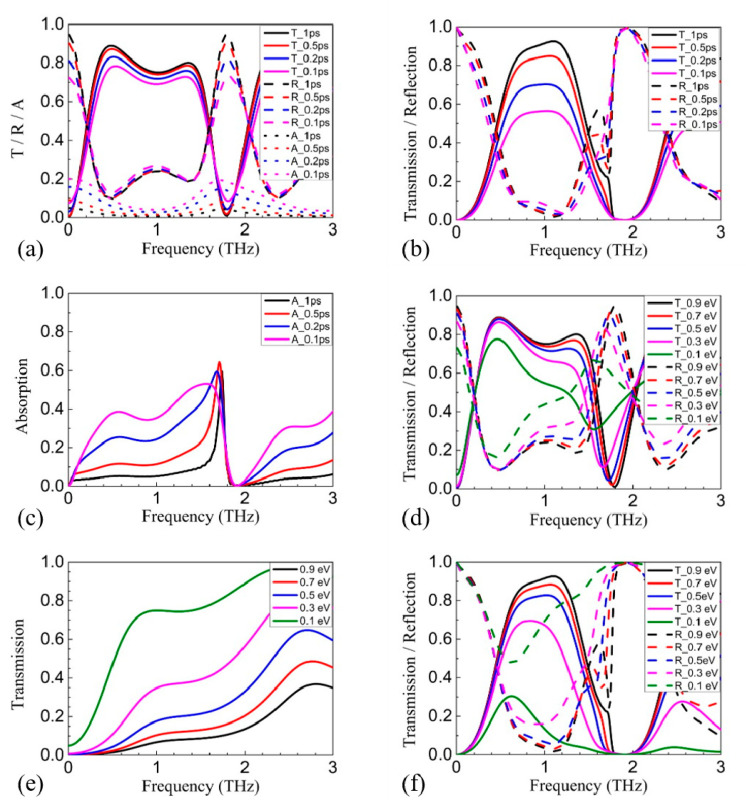
(**a**) The simulated transmission, reflection, and absorption spectra of the v-polarized incidence with only the 2nd GRA pair in the “ON” state and *γ* increased from 10^12^ Hz to 10^13^ Hz. (**b**) The simulated transmission, reflection, and (**c**) absorption spectra of the x-polarized incidence with the 1st and the 2nd GRA pairs in the “ON” state and *γ* increased from 10^12^ Hz to 10^13^ Hz. (**d**) The simulated transmission spectra of the v-polarized incidence with only the 2nd GRA pair in the “ON” state and *E_F_* is reduced from 0.9 eV to 0.1 eV. (**e**) The simulated transmission spectra of the *u*-polarized incidence with *E_F_* reduced from 0.9 eV to 0.1 eV in the absence of the 1st GRA pair, the 3rd GRA pair, and the metallic gratings. (**f**) The simulated transmission spectra of the x-polarized incidence with the 1st and the 2nd GRA pair in the “ON” state and *E_F_* is reduced from 0.9 eV to 0.1 eV.

If the 1st and 2nd GRA pairs are in the “OFF” state and the 3rd pair is in the “ON” state, it can be predicted from the symmetry that the polarization rotation is most efficient for incidence with polarization in the *u*-coordinate. As shown in Figure 3a, the transmission around 0.93 THz exceeds 70% for θ = 20° ~ 65°. Note that the transmission is only ~ 40% for θ ~ 90°, as shown in Figure 2d and Figure 3a. The reason is that the 1st GRA pair is in the “OFF” state and it cannot block the reflected waves. When the 1st GRA pair is in the “ON” state, the Drude absorption causes strong reflection within 2 THz for *y*-polarized electromagnetic waves, as shown by the black curve in Figure 4e, and then the 1st GRA can work as a linear polarizer in the frequency range. Consequently, the proposed structure becomes similar to the one in our previous work [22]. Then, if either the 2nd or the 3rd GRA pair is in the “ON” state, the transmission for θ = 60° ~ 120° is larger than 70%, as shown in Figure 3b,c. Note that the 2nd and the 3rd GRA pair cannot be in the “ON” state simultaneously, or tuning on the phase and the amplitude of the *u*-directional and the *v*-directional electric field components will be the same, which will lead to no polarization conversion. When all the GRAs are in the “OFF” state, the structure contains nothing but *x*-directional gratings, and θ = 0° ~ 30° and 150° ~ 180° can pass through with high transmission for all frequencies, as shown in Figure 2d. Therefore, for arbitrary linear incidence, we can selectively turn on some of the GRA pairs to rotate the incidence to *y*-polarized electromagnetic waves with transmission higher than 70%. 

Since the polarization rotation depends on the tuning on the phase and amplitude by the Drude absorption, the excitation efficiency of the Drude absorption will influence the efficiency of the polarization rotation. Then, it can be inferred that *E_F_* and *γ* will influence the transmission. Figure 4a shows the transmission, reflection, and absorption spectra of the v-polarized incidence with only the 2nd GRA pair in the “ON” state and *γ* increased from 10^12^ Hz to 10^13^ Hz. The increase in *γ* not only weakens the tuning on the phase and the amplitude [22], but also causes more dissipative damping. As a result, both reflection and absorption are increased, and the transmission is reduced by ~ 5%. Figure 4b shows the transmission and reflection spectra of the *x*-polarized incidence with the 1st and 2nd GRA pairs in the “ON” state and *γ* increased from 10^12^ Hz to 10^13^ Hz. Obviously, the transmission decreases by about 30% ~ 40%, while the reflection remains nearly constant. Figure 4c shows that the increase in absorption is the cause of the decrease in transmission. The reason is that the 90° polarization rotation requires more reflections inside the proposed structure, and the amount of lossy material nearly doubles compared to the former case. As a result, the absorption is greatly enhanced. Comparatively, *E_F_* influences the transmission more than *γ*, which is similar to our previous results [22]. The transmission of the *v*-polarized incidence with only the 2nd GRA pair in the “ON” state is reduced by ~ 16% at 0.7 THz and ~ 32% at 1.3 THz when *E_F_* is reduced from 0.9 eV to 0.1 eV, as shown in Figure 4d. Comparatively, the reflection increases by nearly the same amount. The coupling efficiency between the incidence and the free charge carriers in graphene will be reduced as *E_F_* is reduced. Accordingly, the Drude absorption will be weakened, and its broadening will be reduced, as shown in Figure 4e. Consequently, both the tuning in phase and amplitude will be weakened, especially for higher frequencies. For *y*-polarized incidence with the 1st and 2nd GRA pairs in the “ON” state, the influence of *E_F_* is more severe. Apart from the weakened Drude absorption in the 2nd GRA pair, the Drude absorption of the 1st GRA pair is also weakened, which will reduce the reflection on the *y*-polarized waves of the 1st GRA pair within the concerned frequency range, as shown in Figure 4e. Then, the proposed structure gradually becomes dissimilar to the one in our previous work [22] because the blocking of the 1st GRA pair on the *y*-polarized reflected waves from the 2nd GRA pair is greatly reduced, breaking the cavity formed between the 1st and the 2nd GRA pairs. As a result, the reflection increases a lot while the transmission is reduced by ~ 54% at 0.7 THz and ~ 75% at 1.3 THz, as shown in Figure 4f.

**Figure 5:**
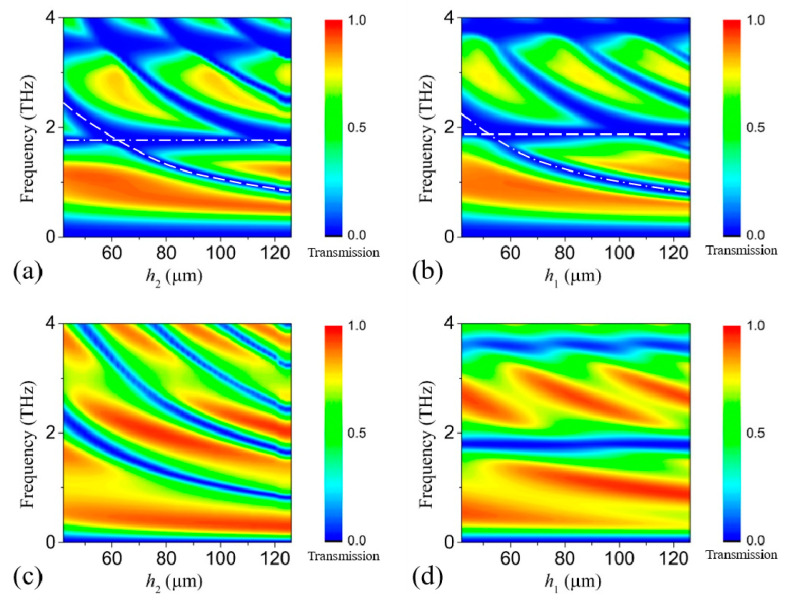
The transmission spectra for *x*-polarized incidence with both the 1st and the 2nd GRA pair in the “ON” state as (**a**) *h*_2_ and (**b**) *h*_1_ increase from 42 μm to 112 μm. The white dashed curve and the white dash-dotted curve indicate the transmission dip caused by *h*_2_ and *h*_1_, respectively. The transmission spectra for *v*-polarized incidence with only the 2nd GRA pair in the “ON” state as (**c**) *h2* and (**d**) *h*_1_ increase from 42 μm to 112 μm.

Finally, we consider the influence of *h*_1_ and *h*_2_ on the transmission. For *x*-polarized incidence with both the 1st and the 2nd GRA pair in the “ON” state, the proposed structure is similar to that in our previous work [22], so that each transmission dip will be divided into two if *h*_1_ ≠ *h*_2_, as shown in Figure 5a,b. For *v*-polarized incidence with only the 2nd GRA pair in the “ON” state, the case is somewhat different. As the *x*-polarized electromagnetic waves will be completely reflected by the metallic gratings, constructive/destructive interaction will take place when *k_0_n_1_h*_2_ is close to an odd/even multiple of π/2, which will enhance/suppress the Drude absorption and thus enhance/suppress the transmission. The corresponding *k*_0_ for constructive/destructive interaction decreases as *h*_2_ increases, inducing the red-shift of the transmission window, as shown in Figure 5c. Comparatively, at the surface of the upper dielectric layer, the reflection for the reflected waves is not so high, so the change in *h*_1_ just slightly influences the transmission. In addition, the phase change is 0 instead of π (at the metallic gratings), so that constructive interaction takes place when *k_0_n_1_h*_1_ is close to even multiple of π/2. Therefore, the transmission at 0.93 THz keeps growing as *h*_1_ increases from 56 μm to 112 μm, as shown in Figure 5d. As the reflection at the surface of the proposed structure is low, the increase at 0.93 THz is only about 20%. In order for the proposed structure to work well for all the linear incidences, *h*_1_ is set equal to *h*_2_.

Finally, we would like to stress that, although GRA pairs are employed as the metamaterial in the simulation, the MMLs can be constituted by other materials. Considering that the *E_F_* influences more than *γ*, few-layer graphene (gated by ion gel) may be better than GRA pairs. Note that the theoretical model just takes the optical conductivity into account, and the model is expected to work for semiconductor spoof surface plasmons [26] or even nanowire arrays [27], which also show a Drude-like dielectric function.

## 4. Conclusions

In this work, we proposed a sandwich structure consisting of an MML on the top, two MMLs extending along the *u*-coordinate and *v*-coordinate in the middle, and metallic gratings extending along the x-coordinate at the bottom. Assuming that GRA pairs constitute the MMLs, for arbitrary linearly polarized incidence, broadband transmission greater than 70% can be obtained by selectively tuning the GRA pairs on or off by electrical bias. The transfer matrix method is applied to theoretically study the polarization rotation. The theoretical analysis and FDTD simulation show that the polarization rotation results from the tuning on the amplitude and phase by the Drude absorption and the cavity effect formed by the upper and lower dielectric separation. A large scattering rate will lead to high absorption, thus reducing the transmission. A low Fermi level will weaken the Drude absorption and its broadening, severely reducing the transmission. In order to enhance the efficiency of the polarization conversion, graphene with high mobility is needed, and the Fermi level or carrier density should also be high. For polarization around 90°, the carrier density of the first MML must be high such that it functions well as a linear polarizer. The proposed structure may find applications in systems which need devices with post-fabrication tunability, such as THz communication, imaging, manipulation, etc.

## Data Availability

The data presented in this study are available on request from the corresponding author.
